# The Impact of Frailty Screening on Radiation Treatment Modification

**DOI:** 10.3390/cancers14041072

**Published:** 2022-02-21

**Authors:** Encarnación Fernández-Camacho, Carlos Ferrer-Ramos, Virginia Morilllo-Macías, Marta Rodríguez-Cordón, Ángel Sánchez-Iglesias, Inmaculada Beato-Tortajada, Alicia Francés-Muñoz, Rodrigo Muelas-Soria, Teresa Piquer-Camañes, Ana Isabel Santafé-Jiménez, Vanessa Aznar-Tortonda, Carlos Ferrer-Albiach

**Affiliations:** Radiation Oncology Service, Hospital Provincial Castellón, Av. del Dr. Clarà 19, 12002 Castelló de la Plana, Spain; encarnacion_90@hotmail.com (E.F.-C.); carlos@gmail.com (C.F.-R.); mrodriguez@comcas.es (M.R.-C.); aalsanchezi@gmail.com (Á.S.-I.); inmabeato@gmail.com (I.B.-T.); alifrances2@hotmail.com (A.F.-M.); rodmuelas@gmail.com (R.M.-S.); teresapiquer.r@gmail.com (T.P.-C.); a.santafe93@gmail.com (A.I.S.-J.); vanessaaznar42@gmail.com (V.A.-T.); ferreralbiach@gmail.com (C.F.-A.)

**Keywords:** oncogeriatry, frailty, therapy modification, Charlson, G8

## Abstract

**Simple Summary:**

Most oncology patients today are ≥65 years, so we should include in our daily practice tools that facilitate the therapeutic approach for elderly patients. Care overburden makes it difficult to perform comprehensive geriatric assessments (CGAs). The aim of our prospective study was to analyze if frailty screening questionnaires, such as G8 or Charlson, could lead to rapid decision making about treatment change in a radiation oncology service. In a homogeneous population of 161 patients, with a median age of 75 years, we found that 28.7% were frail according to the G8 test, while the estimated survival at 10 years was 2.25% based on the Charlson test. The therapeutic modification increased to 21% after frailty analysis, and the radiotherapy prescribed was 5.8 times more likely to be modified in frail patients. Thus, we postulate that the frailty screening test, easier to integrate into clinical practice, is a reliable and efficient aid for optimal approach.

**Abstract:**

Background: Care overburden makes it difficult to perform comprehensive geriatric assessments (CGAs) in oncology settings. We analyzed if screening tools modified radiotherapy in oncogeriatric patients. Methods: Patients ≥ 65 years, irradiated between December 2020 and March 2021 at the Hospital Provincial de Castellón, completed the frailty G8 and estimated survival Charlson questionnaires. The cohort was stratified between G8 score ≤ 14 (fragile) or >14 (robust); the cutoff point for the Charlson index was established at five. Results: Of 161 patients; 69.4% were male, the median age was 75 years (range 65–91), and the prevailing performance status (PS) was 0–1 (83.1%). Overall, 28.7% of the cohort were frail based on G8 scores, while the estimated survival at 10 years was 2.25% based on the Charlson test. The treatment administered changed up to 21% after frailty analysis. The therapies prescribed were 5.8 times more likely to be modified in frail patients based on the G8 test. In addition, patients ≥ 85 years (*p* = 0.01), a PS ≥ 2 (*p* = 0.008), and limited mobility (*p* = 0.024) were also associated with a potential change. Conclusions: CGAs remain the optimal assessment tool in oncogeriatry. However, we found that the G8 fragility screening test, which is easier to integrate into patient consultations, is a reliable and efficient aid to rapid decision making.

## 1. Introduction

One of the main risk factors for the development of tumors is advanced age. According to the American Society of Clinical Oncology (ASCO), 60% of American cancer patients are aged over 65 years [1], with these figures being similar in Spain (60.9%) [2]. Some 22.9% of the Spanish population is aged over 65 years, representing one of the fastest aging European countries in the 20th century [3]. In addition, according to forecasts by the Spanish National Institute of Statistics, this figure will reach 31.4% by 2050, with 11.6% aged over 80 years [4].

The elderly population has more complex health and therapeutic needs. This group implicitly has a lower functional reserve, is more likely to become frail, and is more vulnerable to stressors such as cancer or its treatments [5,6]. These factors, coupled with a lack of evidence, can sometimes lead to the omission of curative treatments, or in contrast, to the application of aggressive treatments [7]. In this sense, the comprehensive geriatric assessment (CGA), which analyzes functionality, comorbidities, polypharmacy, nutritional and psychological status, cognitive ability, and social support has been shown to be an excellent predictor of morbidity and mortality in oncological settings [8,9]. Poor results are associated with greater toxicity after chemotherapy or surgery, greater therapeutic changes, and a poorer quality of life (QoL) [10,11,12].

Although CGA is increasingly being incorporated into medical oncology consultations [13], there is still little dissemination of its use in patients undergoing locoregional treatments [14] because its implementation is resource-heavy, making it difficult to employ in clinical practice. Thus, abbreviated patient-administered versions of the CGA have been developed which can be completed in only a few minutes. Three randomized, clinical trials demonstrated that treatments devised based on the results of these versions improved patient quality of life and toxicity rates without decreasing estimated overall survival [15,16,17].

The G8 test, which was developed specifically for cancer patients, is one of the most widely accepted and most used questionnaires in clinical practice [18,19]. It has been validated in several studies (among which ONCODAGE [20] stands out) and has a high sensitivity to identify patients with a poor prognosis at 1 year (89.6%). A recent systematic review showed an association between the G8 score obtained and the appearance of complications and/or survival after treatment [21]. This tool comprises eight questions that can easily be answered by the patient, either autonomously or with minimal help. An overall G8 score less than or equal to 14 points is considered abnormal.

In contrast, the Charlson comorbidity index does not directly address frailty but was instead developed as a prognostic tool to assess comorbidities that could affect mortality in the short term. Although it was initially created to estimate 1-year survival, this tool was later adapted to estimate 10-year survival [22]. This index comprises 19 medical conditions classified into 4 groups according to the weight (ranging between 1 and 6) assigned to each disease. The sum total represents the relative risk of patient mortality [23]. If the score exceeds 5 points, the probability of mortality is 100%.

Major international societies are now recommending a two-step approach in patients at risk by using a rapid frailty screening tool, followed by CGA in all those aged over 65 years [9,24,25,26]. This current study aimed to analyze the usefulness of systematic implementation of the G8 and Charlson indices in our setting as rapid frailty and survival screening tests during the initial assessment of oncogeriatric patients, as well as to assess the extent of their influence on decisions to modify patient treatments.

## 2. Materials and Methods

This was a descriptive, cross-sectional, observational study with the following three objectives:

1. To describe the characteristics, functional situation, and comorbidities of patients aged over 65 years treated in the Radiation Oncology Service at the Provincial Hospital of Castellón;
2. Analyze the usefulness of the G8 and the Charlson scales in the initial assessment of oncogeriatric patients and the possible influence of these instruments on the modification of our therapeutic approaches;
3. Identify the intercurrent factors most strongly involved in modifying the treatments applied by the physicians in our service.

All patients ≥65 years diagnosed with solid or hematological neoplasia and referred for treatment with ionizing radiation were included in this study. Any patients who declined to participate were excluded. This project was approved by the Ethics Committee at the Castellón Provincial Hospital on 13 September 2019.

All the patients were recruited between December 2020 and March 2021. We provided them with detailed information regarding the study, and then, they gave their written consent for the collection, analysis, and publication of their data as part of this study. At the first visit, the patients completed the frailty and estimated survival questionnaires (the G8 and Charlson indices) as part of their initial anamnesis. The Charlson test is already validated in Spanish [27], while the G8 test has been validated in English, so we translated it to make it easier for patients to understand. 

All of the cases were debated in our medical committee sessions, considering the results obtained, as well as the appropriate therapeutic modifications in each case with respect to the indications published in current guidelines. When the treatment protocol was modified with respect to the guidelines indicated for nongeriatric patients, it was usually to implement a less aggressive procedure with curative intention or to provide exclusively symptomatic management. In addition to the aforementioned questionnaires, demographic and clinical data about the patient age, sex, weight, tumor histology and location, clinical stage, performance status (PS) [28], and treatment received were also collected.

The statistical processing was conducted using SPSS software (version 25.0; IBM Corp., Armonk, NY, USA). First, we conducted a descriptive analysis by assessing the distribution of the variables collected in the sample. We categorized the G8 and Charlson tests to evaluate the association between the score obtained and the modification of the therapeutic approach. Thus, for G8, the patients were stratified according to those who scored equal to or less than 14 (considered as fragile) or those with scores exceeding 14 (healthy or robust). The cutoff point for the Charlson test was established at 5 points, with patients with a value exceeding 5 having an estimated 10-year survival rate of 0%.

The patient characteristics were presented in the form of percentages for qualitative variables and means or ranges for quantitative variables. The normality of the quantitative variables was verified using the Kolmogorov–Smirnoff test. To evaluate our hypothesis, a two-sided *p*-value of less than 0.05 was considered statistically significant. Chi-squared tests were carried out to identify the association between the variables. Once an association was found, its intensity was determined using Cramer’s V, and the ability to predict one variable based on another was verified using the Lambda test. Finally, logistic regression was implemented to ascertain which independent variables influenced the modification of the therapeutic approaches. The variables that showed significant differences in the univariate analysis were further analyzed using multivariate logistic regression analysis.

## 3. Results

### 3.1. Patient and Tumor Characteristics

A total of 161 patients with a median age of 75 years (range 65–91) participated in this study; of those, 69.4% were men, and the predominant PS was 0–1 (83.1%). Only 8.7% reported regular mild-to-moderate alcohol consumption, whereas up to 35% were active smokers at the time of diagnosis (Table 1). The most prevalent tumor location was the prostate (34.4%), followed by the lung (17.5%) and breast (15%). The most frequent histologies were adenocarcinoma and squamous cell carcinoma, with most patients (61.3%) diagnosed as stage II or III.

### 3.2. Charlson and G8 Index Data

More than half of the patients (51.3%) reported comorbidities on the Charlson questionnaire. The most frequent were chronic kidney failure (21.3%), diabetes mellitus (15.63%), and chronic obstructive pulmonary disease (9.3%). The median Charlson score was 6 points (range 4–14), with an estimated median survival of 2.25% at 10 years; in this test, 64.4% of the patients obtained a score exceeding 5 points (Table 2).

Regarding the relevant variables for the G8 test, 71.3% presented a body mass index (BMI) of 23 kg/m^2^ or more, and 3.8% had a BMI of 19 kg/m^2^ or less; in addition, 55% reported involuntary weight loss in the 3 months prior. In total, 20 patients presented reduced mobility that prevented them from leaving their homes, and 70.6% took more than three drugs daily. Despite these data, only 31.3% defined their health status as worse than that of other people their age. Thus, the median G8 score was 13 points (range 2–18), with 28.7% presenting a score of 14 or less, meaning that they were considered fragile (Table 3). 

### 3.3. Treatment Modification

These outcomes resulted in modification of the therapeutic approach in 15.9% of the global sample. It is remarkable that the treatment change increased up to 21% in the subgroup of patients classified as fragile based on the G8 test (*p* = 0.008). We then used contingency tables to identify the variables associated with a potential change in the therapeutic approach. This analysis indicated that the only factors that showed this association were age over 85 years (*p* = 0.01), a PS of 2 or more (*p* = 0.008), and limited mobility (*p* = 0.024).

Regarding geriatric assessment scales, a G8 score of 14 or less was also associated with a change in the treatment approach (*p* = 0.01). However, although a total score of five or more points on the Charlson scale predicted extremely low survival rates, this factor did not appear to be related to modifications to the treatment plans of these patients. The intensity of the associations found was intermediate for age (Cramer’s V = 0.34) but was low for PS (*p* = 0.28), mobility (*p* = 0.22), and the G8 score (*p* = 0.21). Moreover, the Lambda test confirmed these associations but ruled out the possibility of predicting the therapeutic attitude based on these variables. 

Notwithstanding, once we identified which factors showed an association, we analyzed their predictive value by performing regression tests. Thus, it was found that a change in the therapeutic attitude was 11.62 times more likely in patients aged over 85 years. Furthermore, PS1 and PS2 made such changes 5.30 and 12.80 times more likely, respectively. The likelihood of therapeutic modification was 3.38 times higher in patients who had limited mobility but could still leave home than in those with preserved mobility, and 5.07 times higher in those capable of only moving around the house, than that in patients with preserved mobility. Finally, the study showed that, based on the G8 test, a change in treatment was 5.8 times more likely in patients identified as frail than in robust patients.

## 4. Discussion

Aging involves biological and social changes that can influence the evolution and prognosis of oncological diseases. With regard to radiation therapy, those who are already frail may even have difficulty fulfilling some of the technical aspects of treatment [29]. Given that most of our patients are now aged over 65 years, the use of additional tools that allow an individualized therapeutic approach to their healthcare is essential in clinical practice. 

Our study sample is a clear example of this trend, given that in just 3 months, we recruited a cohort of 161 patients that required radiation treatment, with a mean age of 75 years. The analysis of the baseline characteristics allows us to affirm that the sample is representative of the society. For example, our patients present prostate, lung, and breast as the most frequent tumor sites, which matches the latest data from the National Cancer Institute for 2021 [30]. We point out here that the fourth most frequent tumor site, the colon, was not considered in our context due to the lack of indication for radiotherapy for its treatment.

Similarly, we report a high rate of polymedication, with 70.6% of cases taking more than three different medications per day. As stated in a recent review, the percentage is similar in multiple healthcare settings [31]. Polypharmacy has, therefore, become one of the most prevalent and dangerous geriatric syndromes.

Going back to the specific assessment of elderly patients, while comprehensive CGA has proven to be the optimal choice, the time required to complete it makes its use difficult to integrate into everyday clinical practice. Hence, a two-step approach that uses a rapid frailty screening tool in patients aged over 65 years, followed by a comprehensive evaluation in patients who require further study has been recommended by several international societies [9,24,25,26]. 

Thus, in this present study, we selected the G8 frailty and Charlson comorbidity scales as tools for selecting potentially fragile patients. Consistent with other reports in the academic literature [32,33], the time required to complete these questionnaires during consultations was negligible, and therefore, their use did not affect the administration of healthcare on a daily basis. The G8 test highlighted that 28.7% of our patient’s sample was frail, and the Charlson test indicated that the estimated survival at 10 years was 2.25%.

Prior screening of this type has been shown to play a significant role in the selection of therapeutic approaches for patients with lung cancer [34,35], in the prediction of mortality in breast and colon cancer [36,37], the dose and fractionation regimen in radiotherapy protocols [38] as well as fatigue related to the latter [39], and toxicity and tolerance to chemotherapy [40]. 

In this study, we were able to verify how frail patients identified using the G8 scale were 5.8 times more likely to receive treatment other than the standard one. In our scenario, the prescribed radiotherapeutic treatment was modified in up to 21% of the frail population according to the G8 test. Given that a systematic review [41] determined that applying the comprehensive geriatric assessment resulted in a modification to the proposed plan in 28% of cases (usually by adopting a less intensive treatment option), it is remarkable how closely our percentage approaches, using only a screening tool.

Age over 85 years, a PS of 2 or more, an inability to leave home, and a G8 score of 14 or less were the most important variables associated with potential treatment changes in patients in our study population. These factors have already been highlighted in the literature as causes of care changes, so our findings are consistent. Thus, mobility limitations, often associated with multiple chronic conditions, can compromise the quality of care delivery by limiting access to specialists and safe transfer to treatment machines [42]. In the same way, in another study with similar characteristics to ours, but referring to the administration of palliative chemotherapy, functional impairment was associated with increased odds of treatment modification at cycle 1 [43].

Importantly, the literature points to the positive effect of similar interventions, with 75% of the patients in one such study managing to complete treatment with lower rates of complications or toxicity [44]. Although the follow-up time of this current study was insufficient to compare chronic toxicity and survival data, our findings did corroborate a treatment completion rate of 79%. Given these results, we believe that our research opens the possibility of using the G8 test in the future, not as a screening tool prior to CGA but rather as an instrument that could directly indicate the need for treatment modification. Therefore, this research should be continued by expanding the sample size and monitoring patients over longer periods to verify their tolerance to treatments, toxicity, rates of curation, and overall survival.

## 5. Conclusions

The progressive aging of the population leads to an increasing proportion of elderly patients in oncology services. These subjects, who are more likely to be considered “frail”, will require an adjustment of their treatment based on multiple psychosocial, cognitive, functional, polypharmacy, and comorbidity factors.

Although comprehensive geriatric assessment is the optimal evaluation tool for this patient profile, its complexity makes it difficult to integrate it into the currently overburdened healthcare practice.

We found that the G8 fragility screening test, which is easier to integrate into patient consultations, was a reliable and efficient aid to rapid decision making by itself.

## Figures and Tables

**Table 1:** Patient characteristics.

Performance status	0	52 (32.5%)
	1	81 (50.6%)
	2	26 (16.3%)
	3–4	1 (0.6%)
Smoking	Yes	41 (25.6%)
	No	77 (48.1%)
	Unknown	42 (26.3%)
Alcohol habit	Yes	10 (6.3%)
	No	105 (65.6%)
	Unknown	45 (28.1%)
Sex	Male	111 (69.4%)
	Female	49 (30.6%)
Tumor site	Prostate	55 (34.4%)
	Lung	28 (17.5%)
	Breast	24 (15%)
	Head and neck	13 (8.1%)
	Colon/rectum	11 (6.9%)
	Others	29 (18.1%)
Histology	Adenocarcinoma	85 (53.1%)
	Squamous cell carcinoma	34 (21.3%)
	Infiltrating ductal carcinoma	17 (10.6%)
	Others	24 (15%)
Stage	I	23 (14.4%)
	II	45 (28.1%)
	III	50 (31.3%)
	IV	36 (22.5%)
	Unknown	6 (3.8%)
Treatment modification	Yes	24 (15%)
	No	127 (79.4%)
	Unknown	9 (5.6%)

**Table 2:** Data obtained from Charlson index items.

Age (Years)	Median, IQR	75 (65–91)
Stratified age	≥86 years	11 (6.9%)
	80–85 years	44 (27.5%)
	<80 years	105 (65.6%)
Myocardial infarction	Yes	6 (3.8%)
	No	154 (96.2%)
Congestive heart failure	Yes	10 (6.2%)
	No	150 (93.8%)
Peripheral vascular disease	Yes	10 (6.2%)
	No	150 (93.8%)
Cerebrovascular accident	Yes	3 (1.9%)
	No	157 (98.1%)
Dementia	Yes	3 (1.9%)
	No	157 (98.1%)
COPD	Yes	15 (9.4%)
	No	145 (90.6%)
Diabetes mellitus	Yes	20 (12.5%)
	No	140 (87.5%)
Chronic kidney disease	Yes	34 (21.3%)
	No	126 (78.7%)
Charlson score	Median, IQR	6 (4–14)
Stratified Charlson score	0–5	57 (35.6%)
	>5	103 (64.4%)
Estimated 10-year survival	Median, IQR	2.25% (0–53.39)

COPD: chronic obstructive pulmonary disease; IQR: interquartile range.

**Table 3:** Data obtained from G8 index items.

Food intake	Severe decrease	36 (22.5%)
	Moderate decrease	9 (5.6%)
	No decrease	115 (71.9%)
Weight loss	>3 Kg	20 (12.5%)
	1–3 Kg	52 (32.5%)
	No	88 (55%)
Mobility	Bed or chair-bound	7 (4.4%)
	Gets out of bed; does not go out	13 (8.1%)
	Goes out	140 (87.5%)
Body mass index	<19	6 (3.8%)
	19–21	14 (8.8%)
	22–23	26 (16.3%)
	≥24	114 (71.3%)
Drugs per day	0–3	47 (29.4%)
	>3	113 (70.6%)
Health status perception	Worse	50 (31.3%)
	As good	84 (52.5%)
	Better	26 (16.2%)
G8 score	Median, IQR	13 (2–18)
Stratified G8 score	>14	46 (28.7%)
	0–14	114 (71.3%)

## Data Availability

The data that support the findings of this study are available from the corresponding author, V.M.M., upon reasonable request; in order to preserve patient privacy.
